# Supplementary material for: Drug classes affecting intracranial aneurysm risk: Genetic correlation and Mendelian randomization
Source: Eur Stroke J. 2024 Feb 15;9(3):687–95. doi: 10.1177/23969873241234134 (PMC11418413; doi:10.1177/23969873241234134)
Supplement: sj-docx-1-eso-10.1177_23969873241234134 – Supplemental material for Drug classes affecting intracranial aneurysm risk: Genetic correlation and Mendelian randomization [file sj-docx-1-eso-10.1177_23969873241234134.docx]

**STROBE-MR checklist of recommended items to address in reports of Mendelian randomization studies**^1^ ^2^

| **Item No.** | **Section** | **Checklist item** | **Page No.** | **Relevant text from manuscript** |
| --- | --- | --- | --- | --- |
| 1 | **TITLE and ABSTRACT** | Indicate Mendelian randomization (MR) as the study’s design in the title and/or the abstract if that is a main purpose of the study | 1 | Title: Intracranial aneurysm risk and drug class usage: genetic correlation and Mendelian randomization  Author note: MR is not the main purpose, but has an important role in the study. |
|  | **INTRODUCTION** |  |  |  |
| 2 | **Background** | Explain the scientific background and rationale for the reported study. What is the exposure? Is a potential causal relationship between exposure and outcome plausible? Justify why MR is a helpful method to address the study question | 5 | We hypothesize that additional drug classes are present that can help to direct drug discovery. MR is an approach to assess causality of an exposure (in this study, drug usage) on an outcome (ruptured IA, unruptured IA, and the combined group) if specific assumptions are met.^12^ |
| 3 | **Objectives** | State specific objectives clearly, including pre-specified causal hypotheses (if any). State that MR is a method that, under specific assumptions, intends to estimate causal effects | 5-6 | We aimed to identify drug classes affecting the liability to IA to gain insight in the development and/or rupture of IA and potentially identify novel therapeutic mechanisms. For this we used a two-step genetic approach. First, we estimated the extend of genetic overlap between the liability to the usage of drugs within a drug class on the one hand, and IA (unruptured IA and ASAH as separate groups and combined) on the other hand. To identify novel mechanisms, we performed these analyses independent of blood pressure (BP). Next, for drug class correlated with IA independent of BP we leveraged genetic information to identify potential causal effects of usage of drug classes on IA liability, anticipate the effect of the use drugs within a class to treat IA, and assess the causality of the drug indication on IA liability, all using MR |
|  | **METHODS** |  |  |  |
| 4 | **Study design and data sources** | Present key elements of the study design early in the article. Consider including a table listing sources of data for all phases of the study. For each data source contributing to the analysis, describe the following: |  |  |
|  | a) | Setting: Describe the study design and the underlying population, if possible. Describe the setting, locations, and relevant dates, including periods of recruitment, exposure, follow-up, and data collection, when available. |  | *All recruitment details were provided in the original publications.* |
|  | b) | Participants: Give the eligibility criteria, and the sources and methods of selection of participants. Report the sample size, and whether any power or sample size calculations were carried out prior to the main analysis | 7 | We obtained summary statistics of genome-wide association studies (GWAS) of 23 drug usage traits measured in the UK Biobank,^5^ ASAH, unruptured IA, and IA (the combined group of ASAH and unruptured IA),^10^ and BP (<http://www.nealelab.is/uk-biobank>). See Supplementary Table 1 for study details. |
|  | c) | Describe measurement, quality control and selection of genetic variants |  | *See detailed methods* |
|  | d) | For each exposure, outcome, and other relevant variables, describe methods of assessment and diagnostic criteria for diseases |  | *All assessment details were provided in the original publications.* |
|  | e) | Provide details of ethics committee approval and participant informed consent, if relevant | 7 | Only publicly available aggregated data was used with informed consent in place in the original studies. |
| 5 | **Assumptions** | Explicitly state the three core IV assumptions for the main analysis (relevance, independence and exclusion restriction) as well assumptions for any additional or sensitivity analysis | 8 | MR can infer causality of an exposure on an outcome if three assumptions are met: 1. the genetic variants used are associated with the exposure, 2. there are no unmeasured confounders between the exposure and the outcome, and 3. the genetic variants only affect the outcome through the exposure.^12^ |
| 6 | **Statistical methods: main analysis** | Describe statistical methods and statistics used |  |  |
|  | a) | Describe how quantitative variables were handled in the analyses (i.e., scale, units, model) |  | *Blood pressure was standardized using inverse rank normalization, as described by the authors: http://www.nealelab.is/uk-biobank* |
|  | b) | Describe how genetic variants were handled in the analyses and, if applicable, how their weights were selected |  | *See detailed methods* |
|  | c) | Describe the MR estimator (e.g. two-stage least squares, Wald ratio) and related statistics. Detail the included covariates and, in case of two-sample MR, whether the same covariate set was used for adjustment in the two samples | 8-9 | We selected Causal Analysis Using Summary Effect Estimates (CAUSE) as MR method since it models correlated and uncorrelated horizontal pleiotropy, thereby being more robust to reverse causality than other MR methods, and models the presence of unmeasured confounding factors.^17^ We applied CAUSE to assess the effect of genetic liability for drug usage on the susceptibility to IA. In brief, CAUSE tests if a model that includes unmeasured confounders and a causal effect performs better than a model including only unmeasured confounders. In theory, since CAUSE can account for unmeasured confounders, conditioning drug usage summary statistics on BP therefore should not be necessary. However, as additional check we also performed the analysis using the summary statistics for drug usage conditioned on BP.  We performed the following sensitivity analyses for statistically significant MR effects identified with CAUSE: 1. reverse MR with IA as exposure and drug usage an outcome, and 2. other MR methods being generalized summary statistics-based MR (GSMR),^14^ inverse variance weighted MR^18^, weighted mode MR,^19^ and MR-Egger.^20^ |
|  | d) | Explain how missing data were addressed | 10 | Missing SNPs in either the exposure or outcome GWAS were excluded |
|  | e) | If applicable, indicate how multiple testing was addressed | 10 | We set the multiple testing threshold for genetic correlation and main MR analysis at 0.05/20=2.5×10^-3^, where 20 was the number of independent tests obtained according to the method described in the Supplementary Data (Supplementary Figures 1-4). |
| 7 | **Assessment of assumptions** | Describe any methods or prior knowledge used to assess the assumptions or justify their validity | 10 | We performed sensitivity analyses according to an existing framework.^22^ |
| 8 | **Sensitivity analyses and additional analyses** | Describe any sensitivity analyses or additional analyses performed (e.g. comparison of effect estimates from different approaches, independent replication, bias analytic techniques, validation of instruments, simulations) | 7, 9, 10 | To exclude the role of BP, with increased BP being a main risk factor for IA,^6,7^ we conditioned the summary statistics for the 23 drug usage traits on systolic and diastolic BP, using mtCOJO. This approach mimics the use of BP as a covariate in the source GWAS and allows the identification of IA-associated drug classes that present novel therapeutic mechanisms.^14^  We performed the following sensitivity analyses for statistically significant MR effects identified with CAUSE: 1. reverse MR with IA as exposure and drug usage an outcome, and 2. other MR methods being generalized summary statistics-based MR (GSMR),^14^ inverse variance weighted MR^18^, weighted mode MR,^19^ and MR-Egger.^20^  We tested whether genetically predicted drug response was different in persons taking versus not taking the drug using a t-test, to further rule out confounding including collider bias. |
| 9 | **Software and pre-registration** |  |  |  |
|  | a) | Name statistical software and package(s), including version and settings used | 10 | *For CAUSE, GSMR, LDSC, and mtCOJO see detailed methods*  We used the inverse variance weighted MR method implemented in R package TwoSampleMR to assess causality of drug indications on IA and its subtypes. |
|  | b) | State whether the study protocol and details were pre-registered (as well as when and where) |  | NA |
|  | **RESULTS** |  |  |  |
| 10 | **Descriptive data** |  |  |  |
|  | a) | Report the numbers of individuals at each stage of included studies and reasons for exclusion. Consider use of a flow diagram |  | *No individuals were excluded in the study since we only used summary statistics. see Supplementary Table 1 for study details.* |
|  | b) | Report summary statistics for phenotypic exposure(s), outcome(s), and other relevant variables (e.g. means, SDs, proportions) | 7 | We obtained summary statistics of genome-wide association studies (GWAS) of 23 drug usage traits measured in the UK Biobank,^5^ ASAH, unruptured IA, and IA (the combined group of ASAH and unruptured IA),^10^ and BP (<http://www.nealelab.is/uk-biobank>). See Supplementary Table 1 for study details.  For drug response analyses we used the UK Biobank dataset. UK Biobank data are available to bona fide researchers on application at <http://www.ukbiobank.ac.uk/using-the-resource/>. When analyzing drug usage summary statistics in relation to summary statistics for ASAH, unruptured IA, and IA, we excluded UK Biobank samples in the IA analysis to avoid bias due to shared samples. |
|  | c) | If the data sources include meta-analyses of previous studies, provide the assessments of heterogeneity across these studies |  | *NA* |
|  | d) | For two-sample MR:  i.  Provide justification of the similarity of the genetic variant-exposure associations between the exposure and outcome samples  ii.  Provide information on the number of individuals who overlap between the exposure and outcome studies | 7 | When analyzing drug usage summary statistics in relation to summary statistics for ASAH, unruptured IA, and IA, we excluded UK Biobank samples in the IA analysis to avoid bias due to shared samples. |
| 11 | **Main results** |  |  |  |
|  | a) | Report the associations between genetic variant and exposure, and between genetic variant and outcome, preferably on an interpretable scale | NA | *All associations between genetic variant and exposure are publicly available and source studies are reported in supplementary Table 1.* |
|  | b) | Report MR estimates of the relationship between exposure and outcome, and the measures of uncertainty from the MR analysis, on an interpretable scale, such as odds ratio or relative risk per SD difference |  | *See Figures 3 and 4, and the Supplementary Tables* |
|  | c) | If relevant, consider translating estimates of relative risk into absolute risk for a meaningful time period |  | *NA* |
|  | d) | Consider plots to visualize results (e.g. forest plot, scatterplot of associations between genetic variants and outcome versus between genetic variants and exposure) |  | See Figures 3-5. |
| 12 | **Assessment of assumptions** |  |  |  |
|  | a) | Report the assessment of the validity of the assumptions | 11-12 | Although CAUSE can account for unmeasured confounders, we aimed to confirm the independence of BP by using drug usage summary statistics conditioned on BP. Here, we found no evidence for a causal effect of beta-blockers usage on IA (Figure 3B). This indicates that CAUSE did not fully account for confounding by BP, and the correlation between IA and beta-blocker usage is driven by BP.  Sensitivity analyses using additional MR algorithms showed the same directions of effect, and dependence on BP (Supplementary Table 6). |
|  | b) | Report any additional statistics (e.g., assessments of heterogeneity across genetic variants, such as *I^2^*, Q statistic or E-value) |  | *NA* |
| 13 | **Sensitivity analyses and additional analyses** |  |  |  |
|  | a) | Report any sensitivity analyses to assess the robustness of the main results to violations of the assumptions | 12 | Sensitivity analyses using additional MR algorithms showed the same directions of effect, and dependence on BP (Supplementary Table 6). |
|  | b) | Report results from other sensitivity analyses or additional analyses | 10, 12-13 | Although CAUSE can account for unmeasured confounders, we aimed to confirm the independence of BP by using drug usage summary statistics conditioned on BP. Here, we found no evidence for a causal effect of beta-blockers usage on IA (Figure 3B). This indicates that CAUSE did not fully account for confounding by BP, and the correlation between IA and beta-blocker usage is driven by BP.  The effect of genetically predicted antidepressant response on IA was absent in non-users of antidepressant drugs (OR=1.02, 95% CI=0.88-1.18, P=0.80).  Beta-blocker response was associated with increased IA risk in non-users of beta-blockers (1.27, 95% CI=1.06-1.53, P=0.0085) and with similar effect size but large confidence intervals in beta-blocker users (1.24, 95% CI=0.76-2.03, P=0.39), indicating that genetically predicted beta-blocker response was associated with IA through another mechanism than through the effect of beta-blockers.  CMP was consistent with a causal effect on IA using the inverse variance weighted approach (OR=1.63, 95% CI=1.24-2.14, P=4.7×10^-4^, Supplementary Figure 7, Supplementary Table 9). However, no statistically significant effects were found for most sensitivity analyses indicating a pleiotropic relationship between IA and CMP. We confirmed that genetic liability to CMP increased risk of paracetamol usage (Supplementary Table 9). |
|  | c) | Report any assessment of direction of causal relationship (e.g., bidirectional MR) | 12 | Sensitivity analyses using additional MR algorithms showed the same directions of effect, and dependence on BP (Supplementary Table 6). |
|  | d) | When relevant, report and compare with estimates from non-MR analyses |  | *NA* |
|  | e) | Consider additional plots to visualize results (e.g., leave-one-out analyses) |  | *See Figure 5, and Supplementary Figures 5-7* |
| **.** | **DISCUSSION** |  |  |  |
| 14 | **Key results** | Summarize key results with reference to study objectives | 14 | We identified genetic correlations between IA usage of drug classes antidepressant drugs, paracetamol, acetylsalicylic acid, opioid drugs, beta-blockers and drugs for peptic ulcer and gastro-oesophageal reflux disease. For the anti-depressant drugs, we found evidence that its usage influences the risk of IA, although it involves a risk-increasing effect rather than a risk-reducing one. |
| 15 | **Limitations** | Discuss limitations of the study, taking into account the validity of the IV assumptions, other sources of potential bias, and imprecision. Discuss both direction and magnitude of any potential bias and any efforts to address them | 15-16 | We performed thorough attempts to deal with pleiotropy and potential reverse causation. The CAUSE MR method was previously shown to be most robust to confounding by estimating confounders among several MR methods.^17^ However, in our follow-up analyses were showed that BP was in fact the factor driving the genetic overlap between usage of beta-blockers and liability to IA. In the Supplementary Data we provide a detailed discussion of limitations: limits of extrapolating the results, incomplete correction for BP, unbalanced representation of drugs within a class, and correlation between drug classes. |
| 16 | **Interpretation** |  |  |  |
|  | a) | Meaning: Give a cautious overall interpretation of results in the context of their limitations and in comparison with other studies | 16 | In conclusion, we found evidence for a risk-increasing effect of drugs within the anti-depressant drug class on IA, which effect should be further explored in prospective cohort studies. Lastly, for paracetamol, acetylsalicylic acid, opioid drugs, and drugs for peptic ulcer disease and gastro-oesophageal reflux disease, and beta-blockers, we found shared genetic risk underlying IA. Future studies aiming to untangle these shared mechanisms may improve our understanding of the pathogenesis of IA and ASAH, which may in turn identify processes that can be perturbed to affect the liability to IA and ASAH. |
|  | b) | Mechanism: Discuss underlying biological mechanisms that could drive a potential causal relationship between the investigated exposure and the outcome, and whether the gene-environment equivalence assumption is reasonable. Use causal language carefully, clarifying that IV estimates may provide causal effects only under certain assumptions | 14-15 | We found a potential link between antidepressant drug usage and liability to IA and ASAH. Our findings may imply an adverse response to antidepressant drugs. Depression has been linked to stroke liability in women in epidemiological studies.^28^ Since depression is more prevalent in women it may be a factor explaining why the incidence of ASAH is higher in women. The sex-specificity of depression as a risk factor for IA has not yet been studied. Inferring a causal adverse response should be done with caution for several reasons. First, we were unable to identify the drug(s) within the antidepressant drug class that explain the observed increase in IA and ASAH liability, which is an important follow-up step. Second, in theory, responsiveness to antidepressant drugs may instead be explained by a (hidden or unknown) type or cause of depression which in turn may affect IA and ASAH risk. No evidence for such mechanisms currently exists. A prospective cohort study of the effect of depression and antidepressant drug use on IA or ASAH could provide further insight in our findings.  We found a novel pleiotropic link between genetically predicted paracetamol usage and liability to IA. Based on our genetic correlation analysis, liability to the usage of paracetamol is associated with an increased risk of IA. Paracetamol has been proposed to lower body temperature and thereby protect from severe outcome of other stroke types being acute ischemic and hemorrhagic stroke, but this is not yet supported by evidence from a large clinical trial, and no evidence for a role in ASAH has been shown.^29^ Further studies to identify the causal genes underlying the observed pleiotropy between paracetamol and IA may help understand the underlying pathogenic processes. |
|  | c) | Clinical relevance: Discuss whether the results have clinical or public policy relevance, and to what extent they inform effect sizes of possible interventions | 14, 16 | We found a potential link between antidepressant drug usage and liability to IA and ASAH. Our findings may imply an adverse response to antidepressant drugs. (…) Inferring a causal adverse response should be done with caution for several reasons. (…) A prospective cohort study of the effect of depression and antidepressant drug use on IA or ASAH could provide further insight in our findings.  Future studies aiming to untangle these shared mechanisms may improve our understanding of the pathogenesis of IA and ASAH, which may in turn identify processes that can be perturbed to affect the liability to IA and ASAH. |
| 17 | **Generalizability** | Discuss the generalizability of the study results (a) to other populations, (b) across other exposure periods/timings, and (c) across other levels of exposure |  | *See detailed discussion of limitations in the Supplementary Data.* |
|  | **OTHER INFORMATION** |  |  |  |
| 18 | **Funding** | Describe sources of funding and the role of funders in the present study and, if applicable, sources of funding for the databases and original study or studies on which the present study is based | 15 | This project was funded by the Collaboration for New Treatments of Acute Stroke (CONTRAST) consortium (<https://contrast-consortium.nl>).  This project has received funding from the European Research Council (ERC) under the European Union's Horizon 2020 research and innovation program (grant agreement No. 852173). |
| 19 | **Data and data sharing** | Provide the data used to perform all analyses or report where and how the data can be accessed, and reference these sources in the article. Provide the statistical code needed to reproduce the results in the article, or report whether the code is publicly accessible and if so, where | 7 | Only publicly available aggregated data was used with informed consent in place in the original studies. Data generated in this study is available in the Supplement. |
| 20 | **Conflicts of Interest** | All authors should declare all potential conflicts of interest |  | *The authors declare to have no conflicts of interest* |

This checklist is copyrighted by the Equator Network under the Creative Commons Attribution 3.0 Unported (CC BY 3.0) license.

1. Skrivankova VW, Richmond RC, Woolf BAR, Yarmolinsky J, Davies NM, Swanson SA, et al. Strengthening the Reporting of Observational Studies in Epidemiology using Mendelian Randomization (STROBE-MR) Statement. JAMA. 2021;under review.

2. Skrivankova VW, Richmond RC, Woolf BAR, Davies NM, Swanson SA, VanderWeele TJ, et al. Strengthening the Reporting of Observational Studies in Epidemiology using Mendelian Randomisation (STROBE-MR): Explanation and Elaboration. BMJ. 2021;375:n2233.
