# Supplementary material for: Drug classes affecting intracranial aneurysm risk: Genetic correlation and Mendelian randomization
Source: Eur Stroke J. 2024 Feb 15;9(3):687–95. doi: 10.1177/23969873241234134 (PMC11418413; doi:10.1177/23969873241234134)
Supplement: sj-docx-2-eso-10.1177_23969873241234134 – Supplemental material for Drug classes affecting intracranial aneurysm risk: Genetic correlation and Mendelian randomization [file sj-docx-2-eso-10.1177_23969873241234134.docx]

Supplementary Data

For the manuscript entitled ‘*Drug classes affecting intracranial aneurysm risk: genetic correlation and Mendelian randomization.’*

# Detailed methods

## Genetic correlation

LDSC v1.0.1 python3 compatible was used.^1^ Genetic correlation was calculated between each intracranial aneurysm (IA) subtype versus each drug class (conditioned on blood pressure, BP, see the next paragraph). Subtypes were any IA, unruptured IA, and aneurysmal subarachnoid hemorrhage (ASAH). The 23 drug classes are listed in Supplementary Table 1.

For each trait, summary statistics were obtained and preprocessed with the munge_sumstats script implemented in LDSC. Linkage disequilibrium (LD)-score regression was performed using default settings, with LD-scores and weights provided by the LDSC authors: eur_w_ld_chr.

## Conditioning on blood pressure

Conditioning was done using mtCOJO, implemented in GCTA version 1.92.2 beta3.^2^ Summary statistics for inverse rank normalized diastolic BP and systolic BP were obtain from the Neale lab database (<http://www.nealelab.is/uk-biobank>). Trait codes 4079 and 4080 were used, respectively. SNPs were harmonized and allele frequency information was annotated using the haplotype reference consortium reference data release 1.1. MtCojo was run using default setting, conditioning on diastolic BP and systolic BP together, with LD-scores and weights provided by the authors of LDSC (see paragraph “Genetic correlation”).

Conditioned estimates, standard errors, and p-values from mtCOJO were extracted from columns bC, bC_se, and bC_pval. The conditioned summary statistics were the converted to LDSC format using munge_sumstats.py, included in the LDSC release described in paragraph “Genetic correlation”.

## Calculating the number of independent tests

In R v4.2.1 we loaded pairwise genetic correlation estimates for either all IA subtypes (ruptured, unruptured, and combined) or all drug classes, and converted these to a genetic correlation matrix using dcast() implemented in reshape 2 v1.4.4. With the prcomp() function we performed a principal component analysis on the correlation matrix, and used the get_eigenvalue() function to extract eigenvalues. We selected the number of principal components needed to obtain a cumulative explained variance fraction of 99% as the number of independent variables. The number of independent tests was then defined as the number of independent IA subtypes multiplied by the number of independent drug classes.

## Mendelian randomization with CAUSE

In R v3.5.1, package cause v1.2.0.0320 was used to perform Mendelian randomization (MR) with CAUSE.^3^ For each pair of IA subtype and drug class, summary statistics were loaded in R and harmonized. Summary statistics were merged using the function gwas_merge(). Using a fixed seed, set.seed(42), 1 million random SNPs were selected to estimate the CAUSE nuisance parameters. Then, the full set of merged summary statistics were pruned using ld_prune(), with p-value threshold 1×10^-3^, and r^2^ threshold 0.01. CAUSE MR analysis was then performed using these parameters and pruned SNPs, with default settings using cause().

CAUSE compares the goodness of fit of two models: a sharing model and a causal model. The sharing model allows for unmeasured confounders, but no causal effect. The causal model allows unmeasured confounders and a causal effect. The difference is measured in expected log pointwise posterior density (ELPD), and a Z-score is obtained for the difference in ELPD between the models. The Z-score can be compared to a normal distribution to test statistical significance of the difference (null hypothesis being that the sharing model fits at least as well as the causal model). If the causal model is statistically significantly better than the sharing model, the situation is consistent with a causal effect of the exposure on the outcome. If the causal mode is indeed superior to the sharing model, the effect size and direction of the exposure on the outcome can be obtained from the gamma-statistic.

## Mendelian randomization with GSMR

Generalized summary statistics-based MR (GSMR) implemented in GCTA v1.92.2 beta was used to perform MR.^2^ First, summary statistics for IA subtypes and drug class usage were converted to GSMR format. The effective sample size was used as N. European ancestry samples from the 1000 Genomes reference dataset was used as LD reference panel. Then, bi-directional GSMR was performed with the following settings: --gsmr-snp-min 5; --gsmr-direction 2; --clump-r2 0.01; --gwas-thresh 1e-5.

## Mendelian randomization with TwoSampleMR

TwoSampleMR v0.5.6 was used in R v4.2.1 for two purposes.

The first purpose was to assess the effect of drug class usage on the liability for IA and its subtypes. Here, the inverse variance weighted (IVW), weighted mode, and MR-Egger algorithms were applied to deal with potential reverse causation and confounding. A p-value threshold of p<1×10^-5^ and r^2^-threshold of 0.001 was applied to select LD-independent SNPs. Other settings were left as default.

The second purpose of using TwoSampleMR was to do an in-depth assessment of the relationships between IA and indications from drug classes correlated to IA (being pain, peptic ulcer disease, and gastro-oesophageal reflux disease). Summary statistics were loaded, harmonized, and clumped with TwoSampleMR according to the default settings. Variant selection was performed based on the p-value threshold p<5×10^-5^. IVW statistics were used as main outcome, and the weighted mode, weighted median, simple mode, and MR-Egger statistics as sensitivity analyses.

## Drug response Mendelian randomization

To assess the effect of usage of a drug on IA and ASAH, we applied an MR framework according to an earlier study.^4^ Drug users were defined by using any of the products listed in Supplementary Table 2 for that drug (class) during any of the assessments of the UK Biobank. Non-white participants and those with Ehlers-Danlos disease, Marfan syndrome, or autosomal dominant polycystic kidney disease (ICD-10 codes Q87.4, Q79.6, Q61.1, Q61.2, and Q61.3) were excluded. IA patients were defined as having a diagnosis or death record with an ICD-10 code for IA or ASAH (I67.1 or I60) or having a curated diagnosed ASAH listing (phenotype code 42013). ASAH status was based on the previously curated variable (42013), consisting of patients with a diagnosed ASAH. Age at ASAH was obtained from date at ASAH (phenotype code 42012) minus date of birth.

SNPs associated with drug response were obtained from published drug response GWASs: blood pressure response to beta-blockers,^5^ non-treatment-resistant depression (i.e., response to at least one anti-depressant drug),^6^ response to selective serotonin reuptake inhibitors,^6^ and response to antidepressant drugs citalopram and escitalopram.^7^

SNPs with p-values below 5×10^-8^ were included, except for the traits “response to SSRI” and “response to citalopram and escitalopram” for which no SNPs with p<5×10^-8^ were present and a threshold of p<5×10^-5^ was applied. Clumping was performed using the UK Biobank genotypes to extract linkage disequilibrium independent SNPs (r^2^ below 0.001) using plink v1.9 (ref.^8^). Polygenetic scores (PGSs) were calculated in the UK Biobank cohort using plink v2.0 (ref.^9^) option --score with a higher score indicating a higher responsiveness to the drug. For each chromosome the per-allele scores were multiplied by the number of alleles considered, and the PGSs were summed over chromosomes. Tertiles of PGS were determined based on the combined drug user and drug non-user groups. The middle third of PGS was excluded. In the remaining group, among drug users, the effect of PGS tertile on IA/ASAH was calculated using three models:

1. A logistic regression with IA as outcome and the following model:

$$IA \sim PGS+sex+PC1..10+ \varepsilon$$

PC1..10 are the first ten genetic principal components (UK Biobank phenotype code 22009).

1. The same model as 1, but with ASAH as outcome.
2. A Cox proportional hazard model as follows:

$$ASAH survival \sim PGS+sex+PC1..10+ \varepsilon$$

With ASAH survival defined as the age at first ASAH in cases, age at death or age at latest assessment date (the date of the last ASAH in the dataset) as censoring time.

For beta blocker response only one SNPs was included. Therefore, the group was split in two approximately equal groups, which resembled a dominant model with heterozygous and homozygous alternative (non-ancestral) allele carriers having a low PGS and homozygous ancestral individuals having a high PGS.

The analyses were also performed in non-users of the respective drugs. The rationale is that if the effect is only present in drug users, this rules out that pleiotropic pathways explain the observed effect.

# Discussion of limitations

## Why CAUSE did not fully account for confounding and reverse causality.

We selected CAUSE to study the potential causal effects of cardiovascular drug usage on IA liability due to its robustness to reverse causality and pleiotropy. Nevertheless, we found by conditioning drug usage traits on BP that there was in fact a residual confounding factor (namely BP) at play. Furthermore, we observed a risk-increasing effect of cardiovascular disease drug usage on IA. This unexpected finding suggests that reverse causality may be at play. As outlined by the authors of CAUSE, evidence for causality in both directions may be observed if 1) there is a causal effect in one direction that accounts for nearly all the heritability of the descendant, 2) there may be causal effects in both directions, or 3) the traits are closely related and share nearly all genetic variants without a causal relationship. Given that BP is a major causal risk factor for IA,^10, 11^ the observed effects are consistent with a scenario where BP and cardiovascular drug usage are highly related, and BP explains a large proportion of IA heritability.

## Limits to the extrapolation of the results

Here we found genetic overlap between IA and anti-depressant drug use, paracetamol use, acetylsalicylic acid use, opioid drug use, and peptic ulcer and gastro-oesophageal reflux disease drug use. We did not prove causality of the liability to usage of any of the drug classes on IA, but we did find evidence for a risk-increasing effect of antidepressant drug usage on IA, and evidence for shared underlying mechanisms between IA and other drug classes which may be perturbed by existing drugs or informs the identification of new drug targets. The degree of genetic correlation does not allow the estimation of the effective dosage of drugs within the identified classes.

In this study we used genetic summary data from participants of European ancestry. Therefore, the observed correlations may be ancestry-specific and drugs targeting these shared underlying mechanisms may not be effective in all populations.

## Incomplete conditioning on BP

We aimed to exclude the role of BP in correlations and causal relationships between drug usage and IA liability, by conditioning the drug usage summary statistics on BP. To this end, we used summary statistics for BP from the same population as the drug usage traits were obtain from (the UK Biobank). However, BP measurements are performed on a single moment in time and therefore only capture a small portion of variation in BP. The UK Biobank GWAS we utilized explained 15.1% of variance in systolic BP, and 14.3% in diastolic BP (<https://nealelab.github.io/UKBB_ldsc/h2_browser.html>). As a result, some residual BP-related effects may remain after conditioning on BP.

## Unbalanced representation of drugs within a class

Summary statistics for the usage of drugs within a drug class does not imply that all drugs within that class contribute (equally) in the observed genetic overlap between traits. Furthermore, the drug usage summary statistics are driven more by the more commonly taken drugs, and any observed overlap between drug usage and IA liability can be biased toward those drugs. An example is the drugs for peptic ulcer and gastro-oesophageal reflux disease, in which approximately half of the participants took omeprazole.

# Supplementary Figures

**Supplementary Figure 1.** Genetic correlation between drug class usage traits.


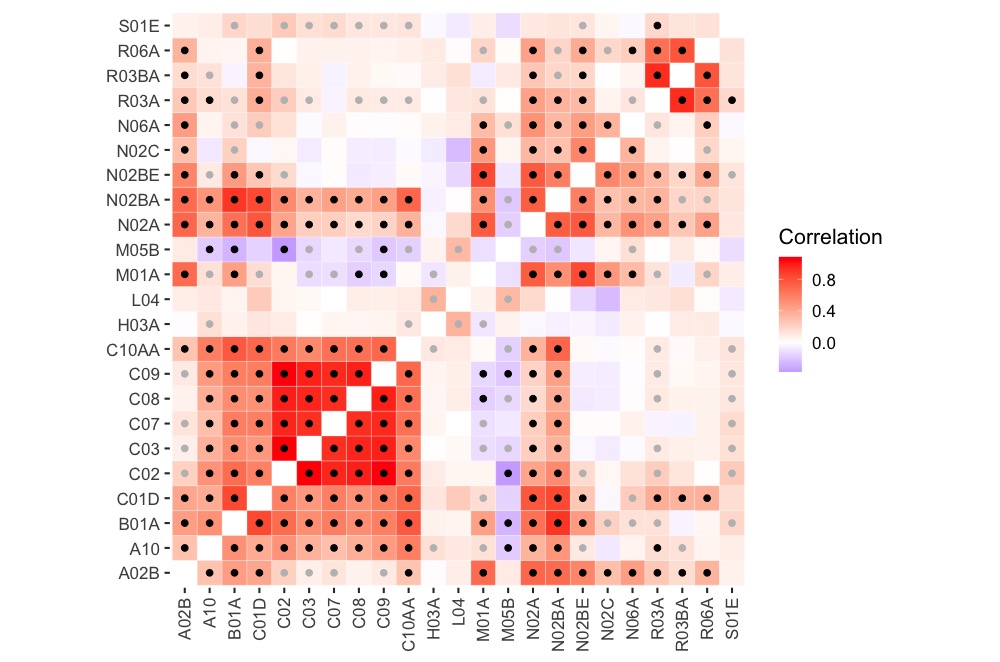


*Color indicates the degree of correlation. Black dots indicate statistical significance (p < 0.05 / 20), while grey dots indicate nominal significance (p < 0.05). Axis labels indicate Anatomical Therapeutic Chemical codes.*

**Supplementary Figure 2.** Scree plot of the first 10 principal components of the genetic correlation matrix of drug classes shown in Supplementary Figure 1.


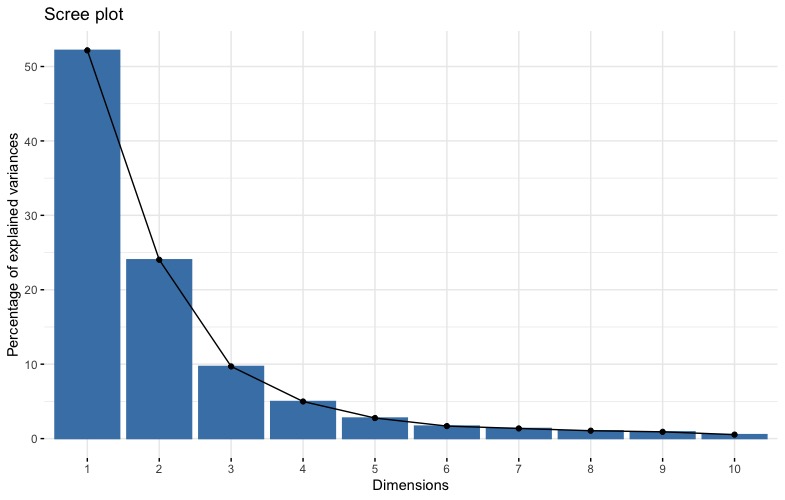


**Supplementary Figure 3.** Genetic correlation between intracranial aneurysm subtypes.


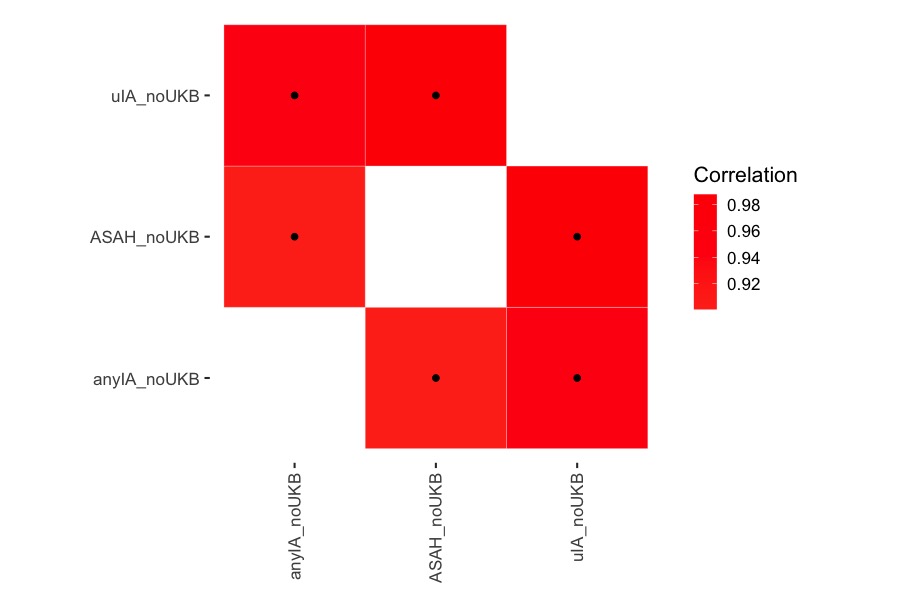


Color indicates the degree of correlation. Black dots indicate statistical significance (p < 0.05 / 20). IA: intracranial aneurysm, UKB: United Kingdom biobank, ASAH: aneurysmal subarachnoid hemorrhage, uIA: unruptured IA.

**Supplementary Figure 4.** Scree plot of the first 10 principal components of the genetic correlation matrix of intracranial aneurysm subtypes shown in Supplementary Figure 3.


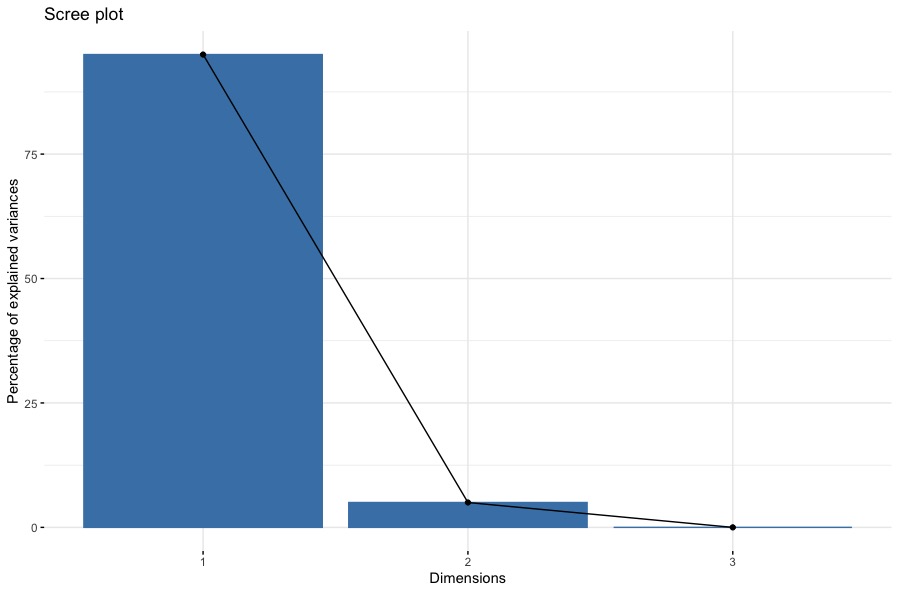


**Supplementary Figure 5.** Drug response Mendelian randomization (MR) analysis with aneurysmal subarachnoid hemorrhage liability as outcome.


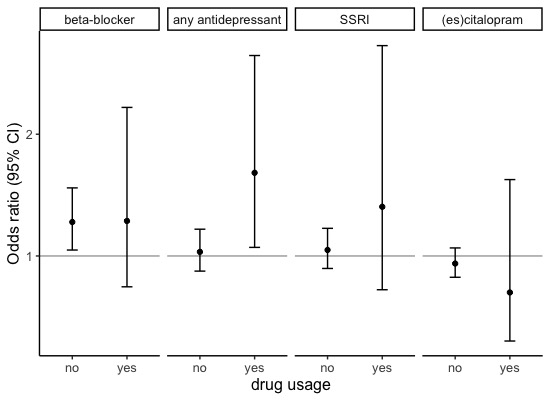


Genetically predicted response to beta-blockers, antidepressant drugs, selective serotonin reuptake inhibitor (SSRI) drugs, and citalopram/escitalopram were selected as exposures, and ASAH was selected as outcome. Per drug, the effect of high versus low predicted response was analyzed in drug users (the group of interest) and non-users (to rule out pleiotropy).

**Supplementary Figure 6.** Drug response Mendelian randomization (MR) analysis with time to aneurysmal subarachnoid hemorrhage as outcome.


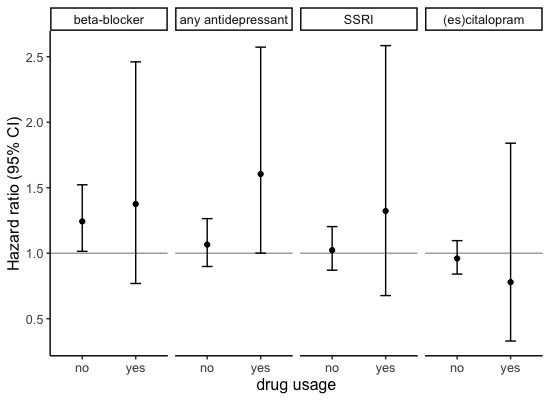


Genetically predicted response to beta-blockers, antidepressant drugs, selective serotonin reuptake inhibitor (SSRI) drugs, and citalopram/escitalopram were selected as exposures, and ASAH risk was selected as outcome. Here, a cox-regression was performed. Per drug, the effect of high versus low predicted response was analyzed in drug users (the group of interest) and non-users (to rule out pleiotropy).

**Supplementary Figure 7.** Mendelian randomization (MR) analysis of drug indications chronic multisite pain (CMP), gastro-oesophageal reflux disease (GORD), and peptic ulcer disease (PUD) as exposure on intracranial aneurysm (IA) liability as outcome.


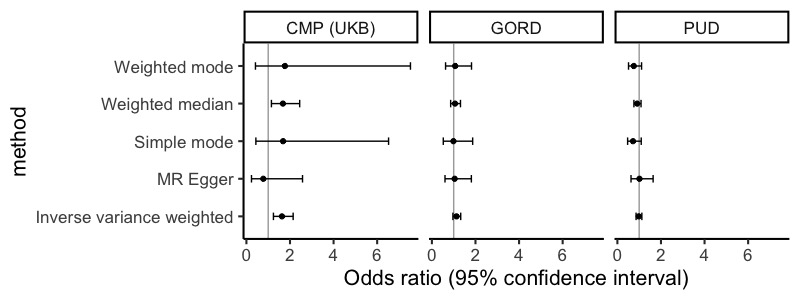


UKB: UK Biobank.

# Supplementary References

1. Bulik-Sullivan B, Finucane HK, Anttila V, et al. An atlas of genetic correlations across human diseases and traits. *Nat Genet* 2015; 47: 1236-1241. 2015/09/29. DOI: 10.1038/ng.3406.

2. Zhu ZH, Zheng ZL, Zhang FT, et al. Causal associations between risk factors and common diseases inferred from GWAS summary data. *Nat Commun* 2018; 9. DOI: ARTN 224

10.1038/s41467-017-02317-2.

3. Morrison J, Knoblauch N, Marcus JH, et al. Mendelian randomization accounting for correlated and uncorrelated pleiotropic effects using genome-wide summary statistics. *Nat Genet* 2020; 52: 740-747. 20200525. DOI: 10.1038/s41588-020-0631-4.

4. Mayerhofer E, Malik R, Parodi L, et al. Genetically predicted on-statin LDL response is associated with higher intracerebral haemorrhage risk. *Brain* 2022; 145: 2677-2686. DOI: 10.1093/brain/awac186.

5. Singh S, Warren HR, Hiltunen TP, et al. Genome-Wide Meta-Analysis of Blood Pressure Response to beta(1)-Blockers: Results From ICAPS (International Consortium of Antihypertensive Pharmacogenomics Studies). *J Am Heart Assoc* 2019; 8: e013115. 20190819. DOI: 10.1161/JAHA.119.013115.

6. Li QS, Tian C, Hinds D, et al. Genome-wide association studies of antidepressant class response and treatment-resistant depression. *Transl Psychiatry* 2020; 10: 360. 20201026. DOI: 10.1038/s41398-020-01035-6.

7. Li QS, Tian C, Seabrook GR, et al. Analysis of 23andMe antidepressant efficacy survey data: implication of circadian rhythm and neuroplasticity in bupropion response. *Transl Psychiatry* 2016; 6: e889. 20160913. DOI: 10.1038/tp.2016.171.

8. Purcell S, Neale B, Todd-Brown K, et al. PLINK: a tool set for whole-genome association and population-based linkage analyses. *Am J Hum Genet* 2007; 81: 559-575. 2007/08/19. DOI: 10.1086/519795.

9. Chang CC, Chow CC, Tellier LC, et al. Second-generation PLINK: rising to the challenge of larger and richer datasets. *Gigascience* 2015; 4: 7. 2015/02/28. DOI: 10.1186/s13742-015-0047-8.

10. Vlak MH, Rinkel GJ, Greebe P, et al. Independent risk factors for intracranial aneurysms and their joint effect: a case-control study. *Stroke* 2013; 44: 984-987. 2013/02/21. DOI: 10.1161/STROKEAHA.111.000329.

11. Bakker MK, van der Spek RAA, van Rheenen W, et al. Genome-wide association study of intracranial aneurysms identifies 17 risk loci and genetic overlap with clinical risk factors. *Nat Genet* 2020; 52: 1303-1313. 2020/11/18. DOI: 10.1038/s41588-020-00725-7.
